# Notch Signaling Activates Yorkie Non-Cell Autonomously in *Drosophila*

**DOI:** 10.1371/journal.pone.0037615

**Published:** 2012-06-05

**Authors:** Hillary K. Graves, Sarah E. Woodfield, Chih-Chao Yang, Georg Halder, Andreas Bergmann

**Affiliations:** 1 Department of Biochemistry and Molecular Biology, The University of Texas MD Anderson Cancer Center, Houston, Texas, United States of America; 2 Graduate Program in Developmental Biology, Baylor College of Medicine, Houston, Texas, United States of America; 3 Program in Genes and Development, The University of Texas MD Anderson Cancer Center, Houston, Texas, United States of America; University of Dayton, United States of America

## Abstract

In *Drosophila* imaginal epithelia, cells mutant for the endocytic neoplastic tumor suppressor gene *vps25* stimulate nearby untransformed cells to express *Drosophila* Inhibitor-of-Apoptosis-Protein-1 (DIAP-1), conferring resistance to apoptosis non-cell autonomously. Here, we show that the non-cell autonomous induction of DIAP-1 is mediated by Yorkie, the conserved downstream effector of Hippo signaling. The non-cell autonomous induction of Yorkie is due to Notch signaling from *vps25* mutant cells. Moreover, activated Notch in normal cells is sufficient to induce non-cell autonomous Yorkie activity in wing imaginal discs. Our data identify a novel mechanism by which Notch promotes cell survival non-cell autonomously and by which neoplastic tumor cells generate a supportive microenvironment for tumor growth.

## Introduction

Imbalances in the cell-cell communication that coordinates cell proliferation, cell differentiation, and cell death can trigger cancer development. Most epithelial cancers arise from single cells that have acquired multiple oncogenic lesions while initially being surrounded by normal cells [1]–[3]. Cell-cell communication between oncogenic cells and surrounding normal cells can create a context that promotes tumor growth and progression.

In *Drosophila,* the genes *avalanche, Rab5, vacuolar protein sorting 25* (*vps25)* and *tumor susceptibility gene 101 (tsg101,* also known as *vps23* and *erupted)* are classified as endocytic neoplastic tumor suppressor genes (nTSGs) because homozygous mutant larvae develop multilayered and invasive tumors with neoplastic characteristics [4]–[9]. Tsg101 and Vps25 are components of the Endosomal Sorting Complex Required for Transport-I (ESCRT-I) and ESCRT-II complexes, respectively, and are necessary to regulate endocytic trafficking of ubiquitylated proteins into internal cellular compartments [10]–[12]. Mutations of *tsg101* or *vps25* cause an endosomal sorting defect resulting in cell-autonomous activation of Notch, Jak/Stat and JNK signaling, loss of apicobasal polarity, and inability to enter a cellular differentiation program [5]–[8]. Nevertheless, when mutant cells of these nTSGs are surrounded by wild-type cells, they undergo JNK-mediated cell death [6], [13]–[15], and only if cell death is blocked, they unleash their tumor-promoting capacity [6], [7].

Unexpectedly, although mutant cells of these nTSGs are highly apoptotic, they are able to non-cell autonomously promote overgrowth of adjacent wild-type tissue before they die. This overgrowth appears to result, at least in part, from altered trafficking of the Notch receptor [5]–[8]. Notch is trapped in abnormal early endsosomes, leading to increased Notch activity as assessed by transcriptional reporters of Notch signaling. Ectopic Notch activation induces increased expression of the secreted cytokine Unpaired (Upd) which stimulates tissue growth in surrounding wild-type cells through activation of the Jak/STAT pathway [5], [7], [8].

In addition to non-cell autonomous overgrowth, our previous studies have shown that *vps25* oncogenic cells can promote non-cell autonomous resistance to apoptotic signals in neighboring cells [6]. This is mediated via non-cell autonomous accumulation of DIAP-1, a potent inhibitor of apoptotic caspases [6], [14]. However, the non-cell autonomous accumulation of DIAP-1 in *vps25* mosaics is not mediated via Upd [6] and has remained unknown. The Hippo/Warts/Yorkie (Hpo/Wts/Yki) pathway is known to control *diap1* expression (reviewed in [16]–[18]). The core components Hpo and Wts negatively regulate the Yki transcription factor through phosphorylation by Wts [16]–[18]. Once Hpo and Wts are inactive, Yki is dephosphorylated and induces target genes such as *diap1* and *expanded* (*ex*). Therefore, we considered the Hpo/Wts/Yki pathway as candidate for non-cell autonomous *diap1* expression in *vps25* mosaics.

Here, we show that activation of Notch, but not of JNK or JAK/STAT, in *vps25* mutant cells can induce non-cell autonomous protection from apoptosis by inducing expression of *diap1*. This increase in DIAP-1 is mediated at the transcriptional level by Yki activity. Additionally, Notch signaling is both necessary and sufficient to induce non-cell autonomous activation of Yki. Therefore, this study identifies a novel mechanism by which Notch signaling can affect growth and cell survival non-cell autonomously in tissues mosaic for mutations in endocytic nTSGs and in general.

## Results and Discussion

### Imaginal Cells Mutant for *vps25* Induce Non-cell Autonomous Yki Activity

**Figure 1:**
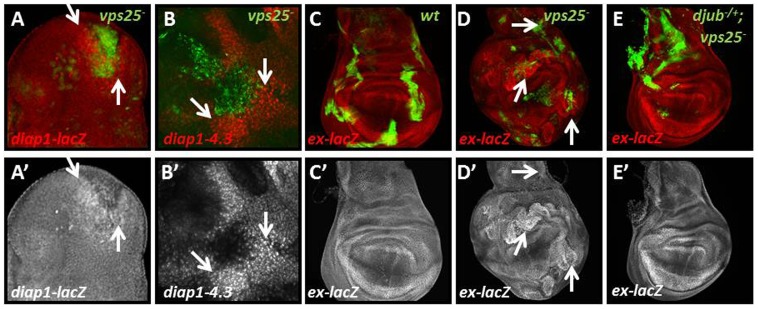
*vps25* mutant cells can induce non-cell autonomous Yorkie activity. Shown are mosaic imaginal discs. Control and *vps25* mutant clones are marked in green. *diap1*-*lacZ* and *ex*-*lacZ* are detected by β-Gal labeling (red or grayscale). Arrows point to representative examples in the panels. (A,A’) *vps25* mutant cells (green) induce non-cell autonomous *diap1*-*lacZ* expression (red and gray) in imaginal discs. (B,B’) *vps25* mutant cells (green) induce non-cell autonomous *Diap1-4.3GFP* (red and grayscale). Please note that GFP is presented in red to match the color code in the other panels. *vps25* mutant clones were identified by ubiquitylation-specific FK1/2 labeling which is known to be increased in *vps25* mutant clones [14] (60×magnification). (C,C’) *ex*-*lacZ* expression (red and grayscale) in wild-type (wt) control mosaic wing discs. (D,D’) *vps25* mutant cells (green) induce non-cell autonomous *ex*-*lacZ* in 71% of wing discs analyzed (n = 28) (red and grayscale). (E,E’) The non-cell autonomous induction of *ex*-*lacZ* (red and grayscale) by *vps25* mutant clones (green) can be suppressed by removing one copy of *djub* (only 29% of wing discs still showed non-cell autonomous *ex-lacZ* (n = 28)). **Genotypes:** (A) *yw hs-FLP; FRT42D Tub-Gal80/FRT42D vps25^N55^ y+; Tub-Gal4, UAS-CD8-GFP/th^j5c8^* (B) *yw hs-FLP; FRT42D piMyc/FRT42D vps25^N55^ y+; Diap1-4.3GFP/+* (C) *yw hs-FLP; FRT42D Tub-Gal80/ex^697^ FRT42D y+; Tub-Gal4, UAS-CD8-GFP/+* (D) *yw hs-FLP; FRT42D Tub-Gal80/ex^697^ FRT42D vps25^N55^ y+; Tub-Gal4, UAS-CD8-GFP/+* (E) *yw hs-FLP/djub^ΔII^; FRT42D Tub-Gal80/ex^697^ FRT42D vps25^N55^ y+; Tub-Gal4, UAS-CD8-GFP/+.*

In *Drosophila* imaginal epithelia, clones of cells mutant for the endocytic nTSG *vps25* can induce neighboring wild-type cells to express DIAP-1 protein [6]. To identify the mechanism by which *vps25* mutant cells regulate DIAP-1 levels in surrounding normal tissue, we first addressed if the accumulation of DIAP-1 is a transcriptional response. Using a *diap1*-*lacZ* reporter, an enhancer-trap insertion that monitors *diap1* transcription [19], we found increased β-Gal labeling surrounding *vps25* mutant cells suggesting a transcriptional response (Figure 1A). Interestingly, the non-cell autonomous expression of *diap1*-*lacZ* is position-dependent and was not observed around every clone. Specifically, in the eye disc this non-cell autonomous effect was more pronounced by *vps25* clones located anterior to the morphogenetic furrow (MF) as compared to posterior to the MF. In the wing disc, *vps25* clones located in the hinge and notum did trigger non-cell autonomous up-regulation of *diap1*-*lacZ*, while clones in the center of the wing pouch did not (see also below).

The non-cell autonomous up-regulation of *diap1*-*lacZ* in *vps25* mosaics suggests a transcriptional response. The Hpo/Wts/Yki pathway is known to transcriptionally regulate *diap1* [20]–[24]. Therefore, we tested for an involvement of the Hpo/Wts/Yki pathway for the non-cell autonomous up-regulation of *diap1* by *vps25* mutant cells by assaying the expression of a *diap1*-*4*.*3GFP* reporter transgene which contains a minimal enhancer responding to Yki [25]. Consistently, we found that *diap1*-*4*.*3GFP* expression was increased non-cell autonomously surrounding clones of *vps25* mutant cells, shown in Figure 1B for the notum of a wing disc, providing evidence that non-cell autonomous induction of *diap1* expression is mediated via Yki activation.

To assay specifically for Yki activity in *vps25* mosaic discs, we examined the expression of *ex*-*lacZ*, a convenient reporter of Yki activity [26], [27]. Compared to controls (Figure 1C), we found that *ex*-*lacZ* was increased non-cell autonomously surrounding patches of *vps25* mutant cells in imaginal discs (Figure 1D). Again, a position-dependence of *vps25* clones was noted, restricting the non-cell autonomous up-regulation of *ex*-*lacZ* to clones in the hinge, some lateral areas of the pouch and notum region of the wing disc, similar to *diap1*-*lacZ*. It has recently been reported that RNAi knockdown of *tsg101* and *vps25* can lead to autonomous Yki activity via JNK signaling [28]. Consistently, we observed autonomous and non-cell autonomous induction of *ex*-*lacZ* when *vps25* was knocked down using RNAi (Figure S1), though autonomous induction of *ex-lacZ* was rarely seen in null *vps25* clones, likely reflecting a difference between hypomorphic and null conditions. Importantly, regardless of the strength of the *vps25* mutation, a non-cell autonomous induction of *ex-lacZ* was consistently observed.

To confirm that the non-cell autonomous increase of *ex*-*lacZ* in *vps25* mosaics is indeed due to Yki activity, we genetically removed one gene dose of a positive regulator of Yki activity, *Drosophila* Ajuba Lim protein (*djub)*, thereby reducing Yki activity [29]. Consistently, heterozygosity of *djub* dominantly suppressed the non-cell autonomous increase of *ex*-*lacZ* seen in *vps25* mosaics (Figure 1E). Taken together, these data suggest that Yki activity is non-cell autonomously increased in wild-type cells adjacent to *vps25* mutant cells, and this increased activity triggers *diap1* transcription promoting non-cell autonomous resistance to apoptosis.

### Blocking Notch Signaling in *vps25* Mutant Cells Suppresses Non-cell Autonomous Induction of Yorkie Activity

**Figure 2:**
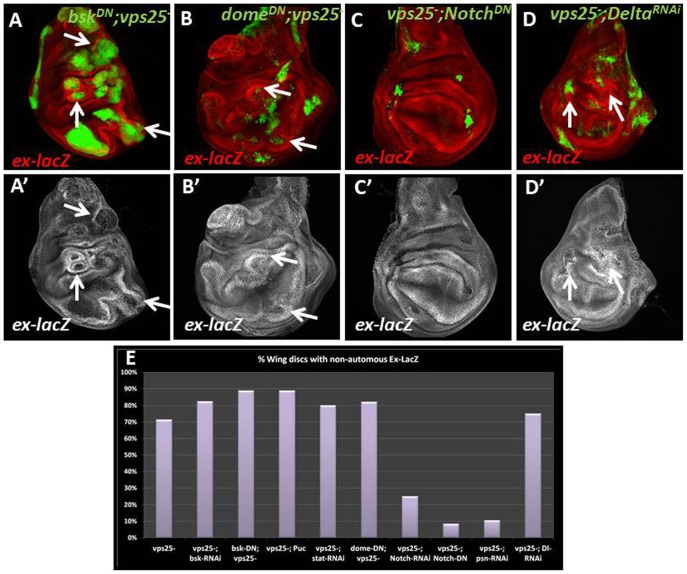
Autonomous inhibition of Notch signaling suppresses non-cell autonomous *ex*-*lacZ* in *vps25* mosaics. Shown are MARCM-induced *vps25* mosaic wing discs expressing the transgenes indicated. *vps25* mutant cells are marked in green. *ex*-*lacZ* is detected by β-Gal labeling (red or grayscale). Arrows point to representative examples. (A,A’,B,B’) Autonomous expression of dominant negative *bsk* (*bsk^DN^*; A,A’) (89% of wing discs showed non-cell autonomous *ex-lacZ* (n = 9)) and *domeless* (*dome^DN^*; B,B’) (82% of wing discs showed non-cell autonomous *ex-lacZ* (n = 28)) does not suppress the non-cell autonomous induction of *ex*-*lacZ* in *vps25* mosaic discs. (C,C’) Autonomous expression of dominant negative *Notch* (*Notch^DN^*) suppresses the non-cell autonomous induction of *ex*-*lacZ* in *vps25* mosaic discs (8% of wing discs showed non-cell autonomous *ex-lacZ* (n = 12)). (D,D’) Autonomous RNAi-induced knockdown of *Delta* (*Delta^RNAi^*) does not suppress the non-cell autonomous induction of *ex*-*lacZ* in *vps25* mosaic discs (75% of wing discs showed non-cell autonomous *ex-lacZ* (n = 12)). (E) Summary of the effects on non-cell autonomous *ex*-*lacZ* when JNK, Jak/STAT or Notch activity are autonomously inhibited in *vps25* mosaic wing discs. At least 10 discs were assayed/genotype. **Genotypes:** (A,B) *yw hs-FLP/UAS-bsk^DN^* or *UAS-dome^DN^*; *FRT42D Tub-Gal80/ex^697^ FRT42D vps25^N55^ y+; Tub-Gal4, UAS-CD8-GFP/+* (C) *yw hs-FLP; FRT42D Tub-Gal80/ex^697^ FRT42D vps25^N55^ y+; Tub-Gal4, UAS-CD8-GFP/UAS- Notch^DN^* (D) *yw hs-FLP; FRT42D Tub-Gal80/ex^697^ FRT42D vps25^N55^ y+; Tub-Gal4, UAS-CD8-GFP/UAS-Delta^RNAi^.*

The signaling events that occur between oncogenic and normal cells in an epithelium are largely unknown. In order to determine which signaling pathways could mediate the non-cell autonomous activation of Yki signaling in *vps25* mosaics, we inhibited pathways known to be activated within *vps25* mutant cells in wing and eye imaginal discs and assayed for effects on non-cell autonomous induction of the *ex*-*lacZ* reporter. JNK signaling is active in *vps25* mutant cells (Figure S2A) and mediates autonomous apoptosis of *vps25* mutant cells [6], [15]. However, autonomous inhibition of the JNK pathway using RNAi to the *Drosophila* JNK ortholog *basket* (*bsk)*, expression of a dominant negative form of Bsk, or overexpression of an inhibitor of the JNK pathway, *puckered* (*puc*), did not block non-cell autonomous *ex*-*lacZ* in *vps25* mosaics (Figure 2A and E). In contrast, due to inhibition of JNK-induced apoptosis under these conditions, *vps25* mutant clones are larger and the non-cell autonomous induction of *ex*-*lacZ* is even more clearly visible. These observations suggest that JNK activation in *vps25* clones does not play a role for non-cell autonomous activation of Yki signaling.

Jak/STAT signaling is thought to mediate non-cell autonomous overgrowth surrounding *vps25* mutant cells [6]–[8]. However, we also detect strong autonomous labeling in *vps25* clones using the phosphoSTAT antibody that detects phosphorylated and thus activated STAT92 protein (Figure S2B). Therefore, we tested for an autonomous involvement of Jak/STAT signaling for the non-cell autonomous control of *ex*-*lacZ* in *vps25* mosaics. However, autonomous reduction of Jak/STAT signaling either by RNAi to *stat92E,* which encodes the transcription factor in the Jak/STAT pathway, or expression of a dominant-negative form of Domeless (*dome^DN^*), the receptor of the pathway, did not inhibit the non-cell autonomous activation of Yki signaling in *vps25* mosaics (Figure 2B and E).

Finally, Notch signaling is known to be ectopically activated in *vps25* mutant cells [6]–[8] as verified by strong up-regulation of the Notch signaling reporter *Gbe-Su(H)-lacZ* (Figure S2C). In contrast to Jak/STAT and JNK signaling, autonomous inhibition of Notch signaling in *vps25* clones by expression of a dominant negative form of Notch in *vps25* clones lead to suppression of non-cell autonomous expression of *ex*-*lacZ* (Figure 2C). Similarly, *Notch* RNAi caused a dramatic decrease in the number of clones that displayed non-cell autonomous induction of *ex*-*lacZ* in *vps25* mosaics (summarized in Figure 2E). This was also true when RNAi to *presenilin*, a positive regulator of Notch signaling, was expressed in *vps25* mutant cells (Figure 2E). Interestingly, the activation of Notch and induction of *ex*-*lacZ* in *vps25* mosaics appear to be ligand independent, as RNAi to the Notch ligand Delta had no effect on the penetrance of non-cell autonomous *ex*-*lacZ* in *vps25* mosaic wing discs (Figure 2D and E) although knock-down was effective because Delta protein is lost in *Delta*-RNAi clones (data not shown). This ligand-independent control of Notch activation in *vps25* mutants is consistent with previous findings obtained in S2 cultured cells [7]. Taken together, these data suggest that ligand-independent Notch signaling from within *vps25* neoplastic cells induces Yki activity in adjacent, wild-type cells.

### Notch is Sufficient to Induce Non-cell Autonomous Yorkie Activity

**Figure 3:**
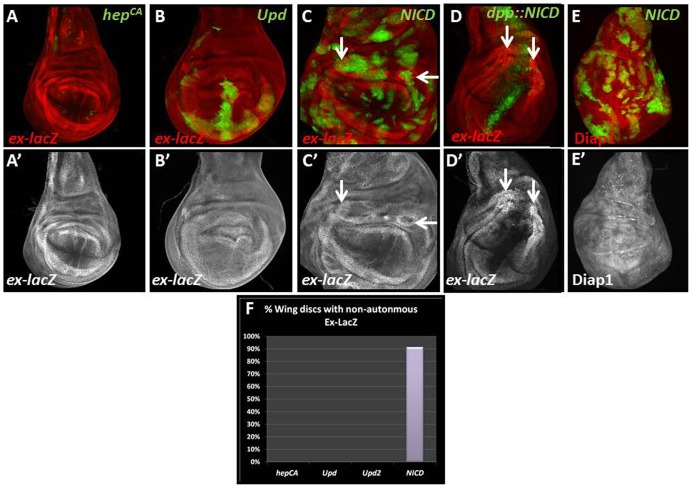
Activation of Notch signaling is sufficient to induce Yorkie activity. Shown are MARCM-induced mosaic wing discs (A,B,C, E) expressing the indicated transgenes in otherwise wild-type clones marked in green. (D) expresses *NICD* under *dpp*-*Gal4*. *ex*-*lacZ* is detected by β-Gal labeling (red or grayscale). Arrows point to representative examples. (A,A’,B,B’) Overexpression of *hep^CA^* (0% of wing discs showed non-cell autonomous *ex-lacZ* (n = 13)) and *upd* (0% of wing discs showed non-cell autonomous *ex-lacZ* (n = 11)) does not lead to non-cell autonomous induction of *ex*-*lacZ*. (C,C’) Overexpression of *NICD* leads to non-cell autonomous induction of *ex*-*lacZ* (91% of wing discs showed non-cell autonomous *ex-lacZ* (n = 23)). (D,D’) Expression of *NICD* using *dpp*-*Gal4* induces strong non-cell autonomous upregulation of *ex*-*lacZ* in the hinge and notum, but not the wing pouch. (E,E’) Overexpression of *NICD* leads to non-cell autonomous accumulation of Diap1 protein. (F) Summary of the effects on non-cell autonomous *ex*-*lacZ* when JNK, Jak/STAT or Notch activity are autonomously induced in wild-type mosaic wing discs. At least 10 discs were assayed/genotype. **Genotypes:** (A,B) *yw hs-FLP; FRT42D Tub-Gal80/ex^697^FRT42D y+; Tub-Gal4, UAS-CD8-GFP/UAS- hep^CA^ or UAS-upd* (C) *yw hs-FLP; FRT42D Tub-Gal80/ex^697^FRT42D y+; Tub-Gal4, UAS-CD8-GFP UAS-NICD* (D) *ex*-*lacZ*; *dpp*-*Gal4 UAS*-*GFP*/*UAS*-*NICD* (E) *yw hs-FLP; FRT42D Tub-Gal80/ex^697^FRT42D y+; Tub-Gal4, UAS-CD8-GFP/UAS-NICD.*

Next, we sought to determine if autonomous hyperactivation of JNK, Jak/STAT or Notch signaling in otherwise wild-type cells is sufficient to induce non-cell autonomous expression of *ex*-*lacZ*. First, mosaic overexpression of a constitutively active form of *hemipterous* (*hep^CA^*), the *Drosophila* JNKK ortholog, yielded very small clones due to JNK-induced apoptosis. However, the few clones that did survive showed no apparent effect on *ex*-*lacZ* (Figure 3A and F). Second, we expressed the ligands Upd and Upd2 to ectopically activate Jak/STAT signaling and saw no effect on *ex*-*lacZ* (Figure 3B and F). These results suggest that the JNK and Jak/STAT signaling pathways are not sufficient to induce non-cell autonomous Yki activity.

Finally, we tested if activation of Notch is sufficient to induce non-cell autonomous expression of *ex*-*lacZ* and thus activity of Yki. Indeed, expression of the activated form of Notch, the intracellular domain (NICD), is sufficient to induce non-cell autonomous expression of *ex*-*lacZ* in wing imaginal discs (Figure 3C and F). Similar to *diap1*-*lacZ* and *ex*-*lacZ* expression in *vps25* mosaics, we noted a position-dependence of *NICD*-expressing clones for *ex*-*lacZ* expression. To further characterize the position-dependence, we expressed *NICD* using *dpp*-*Gal4* along the anterioposterior axis of the wing disc. Consistently, strong non-cell autonomous *ex*-*lacZ* expression is observed in the hinge region (see arrows in Figure 3D and D’). In contrast, expression of *NICD* in the center of the wing pouch does not cause non-cell autonomous *ex*-*lacZ* expression. Finally, *NICD* expression was also able to induce non-cell autonomous accumulation of DIAP-1 (Figure 3E), suggesting that Notch can control cell survival non-cell autonomously.

In summary, our study identifies a novel role of Notch signaling for non-cell autonomous control of apoptosis via induction of Yki activity in neighboring cells. It has previously been shown that Notch signaling controls cell proliferation both autonomously and non-cell autonomously in the developing eye [30]. The non-cell autonomous component of proliferation control was attributed to Notch-dependent activation of Jak/Stat signaling [31] although that recently came into question [32]. Nevertheless, Jak/Stat activation is not sufficient to mediate the effect of Notch on non-cell autonomous control of apoptosis [6] (Figure 3B,E). Here, we identify the Hpo/Wts/Yki pathway as a target of Notch signaling for the non-cell autonomous control of apoptosis both in eye and wing imaginal discs. Because the Hpo/Wts/Yki pathway also controls proliferation, it is likely that Notch promotes non-cell autonomous proliferation through both Jak/Stat and Hpo/Wts/Yki activities.

It is also interesting to note that this non-autonomous control of the Hpo/Wts/Yki pathway by Notch occurs in a position-dependent manner. For example, *vps25* mutant clones or *NICD*-expressing clones located in the hinge and notum of wing discs triggered non-cell autonomous up-regulation of *ex*-*lacZ*, while clones in the wing pouch did not (Figure 1D, 3D). Additionally, *vps25* mutant clones located anterior to the morphogenetic furrow triggered non-cell autonomous up-regulation of *ex*-*lacZ*, while clones in the posterior of the eye disc did not. The reason for this position-dependence is unknown. However, the regions which do not induce Hpo/Wts/Yki signaling non-autonomously correspond to the zone of non-proliferating (ZNP) cells in the wing disc and post-mitotic, differentiating cells in the eye disc [33]. Therefore, one potential reason for the position-dependence may be that the post-mitotic nature of the cells in the ZNP of the wing pouch and in the posterior of the eye disc render them inert to growth-promoting signals that trigger the Hpo/Wts/Yki pathway. However, while this is one possibility, there may also be additional mechanisms that influence the response to growth-promoting signals.

How Notch exerts this non-autonomous effect is an important and interesting question. Based on its function as a transcriptional regulator, it is possible that increased Notch signaling in *vps25* mutant cells could lead to transcription of a secreted or transmembrane protein that communicates to surrounding tissue and induces Yki activity. Expression of proteins known to non-cell autonomously activate Yki signaling such as Fat, Dachsous (Ds) and Four-Jointed as well as *ds-lacZ* (reviewed in [16]–[18]), however, are not altered in *vps25* mosaic discs (data not shown). Identification of this non-cell autonomous signaling mechanism may also be critical for understanding tumorigenesis, as mutations in the Notch pathway, the Hippo pathway and in ESCRT components have been implicated in many different types of human cancer (reviewed in [17], [34]–[37]). In conclusion, this study provides a mechanism by which neoplastic cells influence the behavior of neighboring wild-type cells, which may be critical for generating a supportive microenvironment for tumor growth by preventing cell death and promoting the proliferation of wild-type cells.

## Materials and Methods

### Fly Stocks

The following mutants and transgenic lines were used: *vps25^N55^* [6]; *djub^ΔII^* [29]; *diap1*-*lacZ* = *th^j5c8^* [19]; *diap1*-*4*.*3GFP* [25]; *ex*-*lacZ* = ex^697^ [26]; *UAS*-*bsk^DN^* [38]; *UAS*-*dome^DN^* [39]; *UAS*-*NICD* and *UAS*-*Notch^DN^* [40]; *UAS*-*Notch^RNAi^* (VDRC); *UAS*-*Delta^RNAi^* (VDRC); *UAS*-*psn^RNAi^* (VDRC); *UAS*-*vps25^RNAi^* (VDRC); *UAS*-*hep^CA^* [41]; *UAS*-*upd* [42]; *UAS*-*upd2* [43]; *puc*-*lacZ* [44]; *dpp*-*Gal4* and *ptc-Gal4* (Bloomington); *Su*(*H*)-*Gbe-lacZ* [45].

### Mosaics

Mosaics were generated using the MARCM (mosaic analysis using a repressible cell marker) technique which allows expression of transgenes such as *UAS-GFP* in mutant clones [46]. Heat shocks were administered for 1 hour at 37°C at 48 and 72 hours after egg laying to induce clones.

### Immunohistochemistry

Imaginal discs were dissected from 3^rd^ instar larvae and stained using standard protocols. The following antibodies were used: mouse α-βGal (1∶500; DSHB), guinea pig α-DIAP1 (1∶1000; kind gift of Pascal Meier), and rabbit pStat (1∶100; Cell Signaling Technology). Cy3-conjugated anti-guinea pig and anti-mouse (Jackson ImmunoResearch) were used as secondary antibodies. Images were obtained using an OlympusFV500 confocal microscope and processed using Adobe Photoshop CS4.

## Supporting Information

Figure S1 ***vps25*** **RNAi induces autonomous and non-cell autonomous induction of** ***ex*** **-** ***lacZ*** **(related to** **Figure 1** **).** *vps25* was knocked down by RNAi along the anteroposterior boundary using *ptc*-*Gal4* (grey in A”). (A) is the merged image of GFP (*ptc*-*Gal4*) and red (*ex*-*lacZ*). Yellow stippled lines in (A’) indicates the *ptc*-*Gal4* expression domain based on (A”). Both autonomous and non-cell autonomous (white arrow) expression of *ex*-*lacZ* is detectable. However, in the center of the wing pouch area (green arrow) neither autonomous nor non-cell autonomous *ex*-*lacZ* is induced, suggesting position-dependence of the location of *vps25* inhibition on *ex*-*lacZ* induction. **Genotype**: *ex*-*lacZ ptc*-*Gal4 UAS*-*GFP*; *UAS*-*vps25^RNAi^*
(TIF)

Figure S2 **JNK, Jak/STAT and Notch signaling are activated in** ***vps25*** **mutant cells (related to** **Figure 2** **).** Shown are MARCM-induced *vps25* mosaic wing discs with the indicated gene reporters. *vps25* mutant cells are marked in green. *puc*-*lacZ* (A) and *Su(H)-Gbe-lacZ* (C) are detected by β-Gal labeling (red or grayscale). Arrows point to representative examples. (A,A’) *puc*-*lacZ* is increased in *vps25* mutant clones. (B,B’) Phosphorylated Stat (pStat) protein (red and grayscale) is increased in *vps25* mutant clones. (C,C’) *Su(H)-Gbe-lacZ* is increased in *vps25* mutant clones. **Genotype**s: (A) *yw hs-FLP; FRT42D Tub-Gal80/FRT42D vps25^N55^ y+; Tub-Gal4, UAS-CD8-GFP/puc-lacZ* (B) *yw hs-FLP; FRT42D Tub-Gal80/FRT42D vps25^N55^ y+; Tub-Gal4, UAS-CD8-GFP/+* (C) *yw hs-FLP; FRT42D Tub-Gal80/FRT42D vps25^N55^ y+; Tub-Gal4, UAS-CD8-GFP/Su(H)-Gbe-lacZ*
(TIF)
