# Supplementary figures and images for: Notch Signaling Activates Yorkie Non-Cell Autonomously in Drosophila
Source: PLoS One. 2012 Jun 5;7(6):e37615. doi: 10.1371/journal.pone.0037615 (PMC3367968; doi:10.1371/journal.pone.0037615)

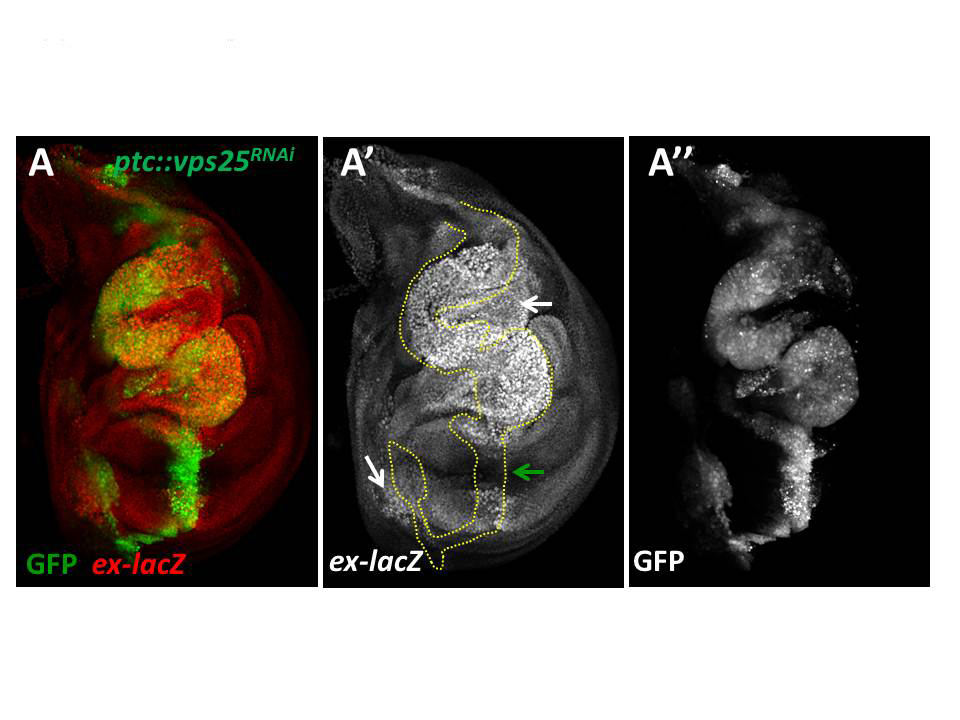

Supplement: Figure S1 — vps25 RNAi induces autonomous and non-cell autonomous induction of ex - lacZ (related to Figure 1 ). vps25 was knocked down by RNAi along the anteroposterior boundary using ptc-Gal4 (grey in A”). (A) is the merged image of GFP (ptc-Gal4) and red (ex-lacZ). Yellow stippled lines in (A’) indicates the ptc-Gal4 expression domain based on (A”). Both autonomous and non-cell autonomous (white arrow) expression of ex-lacZ is detectable. However, in the center of the wing pouch area (green arrow) neither autonomous nor non-cell autonomous ex-lacZ is induced, suggesting position-dependence of the location of vps25 inhibition on ex-lacZ induction. Genotype: ex-lacZ ptc-Gal4 UAS-GFP; UAS-vps25RNAi (TIF) [file pone.0037615.s001.tif]

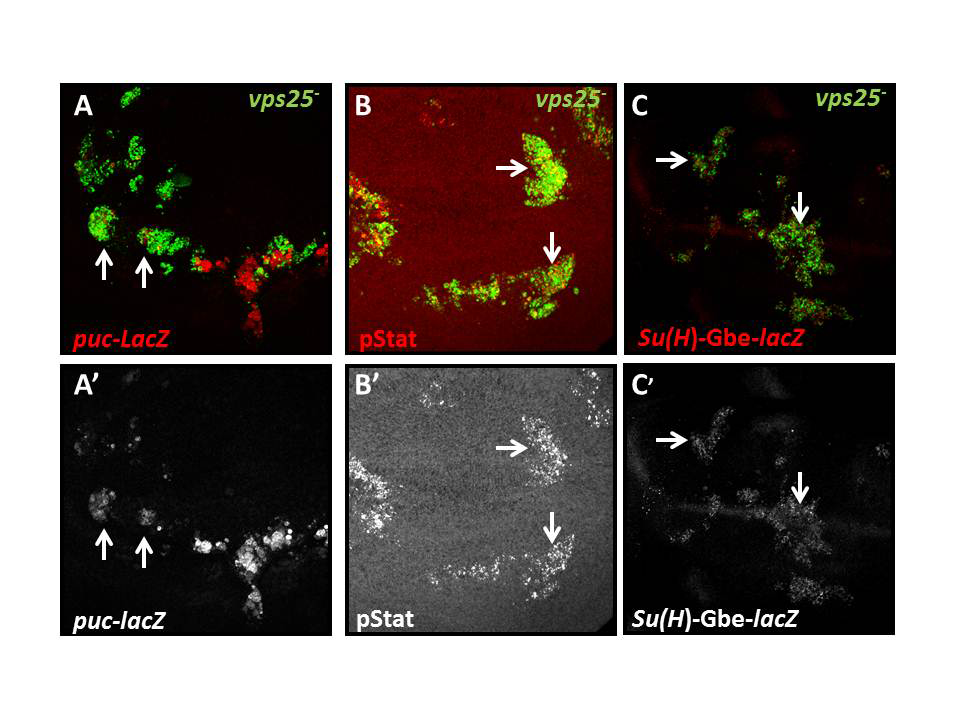

Supplement: Figure S2 — JNK, Jak/STAT and Notch signaling are activated in vps25 mutant cells (related to Figure 2 ). Shown are MARCM-induced vps25 mosaic wing discs with the indicated gene reporters. vps25 mutant cells are marked in green. puc-lacZ (A) and Su(H)-Gbe-lacZ (C) are detected by β-Gal labeling (red or grayscale). Arrows point to representative examples. (A,A’) puc-lacZ is increased in vps25 mutant clones. (B,B’) Phosphorylated Stat (pStat) protein (red and grayscale) is increased in vps25 mutant clones. (C,C’) Su(H)-Gbe-lacZ is increased in vps25 mutant clones. Genotypes: (A) yw hs-FLP; FRT42D Tub-Gal80/FRT42D vps25N55 y+; Tub-Gal4, UAS-CD8-GFP/puc-lacZ (B) yw hs-FLP; FRT42D Tub-Gal80/FRT42D vps25N55 y+; Tub-Gal4, UAS-CD8-GFP/+ (C) yw hs-FLP; FRT42D Tub-Gal80/FRT42D vps25N55 y+; Tub-Gal4, UAS-CD8-GFP/Su(H)-Gbe-lacZ (TIF) [file pone.0037615.s002.tif]
